# *Rhodnius micki*, a new species of Triatominae (Hemiptera, Reduviidae) from Bolivia

**DOI:** 10.3897/zookeys.1012.54779

**Published:** 2021-01-26

**Authors:** Yisheng Zhao, Cleber Galvão, Wanzhi Cai

**Affiliations:** 1 Department of Entomology and MOA Key Lab of Pest Monitoring and Green Management, College of Plant Protection, China Agricultural University, Beijing 100193, China China Agricultural University Beijing China; 2 Laboratório Nacional e Internacional de Referência em Taxonomia de Triatomíneos, Instituto Oswaldo Cruz, LNIRTT/IOC/FIOCRUZ, Pavilhão Rocha Lima, 5° andar, Avenida Brasil, 4365, Manguinhos, RJ, Brazil Instituto Oswaldo Cruz Rio de Janeiro Brazil

**Keywords:** Comparative terminology, genitalia, geometric morphology, kissing bug, taxonomy

## Abstract

*Rhodnius* Stål, 1859 is the second largest genus of Triatominae after *Triatoma* Laporte, 1832, and includes several important Chagas vectors. Genitalia in Reduviidae are frequently used for species identification, but the current use of terminology for it is inconsistent in Triatominae. Here, *Rhodnius micki***sp. nov.**, is described from Bolivia and considered as belonging to the *pictipes* group based on its morphological characters and distribution. Detailed documentation of the genitalia of *Rhodnius micki***sp. nov.** is provided with emphasis on its everted phallus, especially the endosomal sclerites, which are potentially useful as species-level diagnostic features in *Rhodnius*. To further verify the validity of this species, the head shapes and wing venation patterns of five species in *Rhodnius* are compared with morphometric analysis. After reviewing taxonomic and comparative morphology papers of assassin bugs, a vocabulary with a terminology of morphological characters, especially of external male genitalic characters, is assembled with the preferred terms and the synonyms listed. Establishing a consistent terminological framework will greatly facilitate future research on the homology of these structures across Triatominae and will ultimately contribute to our understanding of the evolution of these groups.

## Introduction

Triatominae are a subfamily within Reduviidae that is known for its hematophagous feeding habit (Jansen and Roque 2010). Currently, there are 151 extant and three known fossil species assigned to 18 genera and five tribes in Triatominae (Lent and Wygodzinsky 1979; Justi and Galvão et al. 2017; Rosa et al. 2017a; Oliveira et al. 2018; Lima-Cordón et al. 2019; Nascimento et al. 2019; Poinar Jr 2019). All Triatominae possess a nearly straight labium with a flexible membranous connection between the second and third visible segments that allows upward pointing when feeding (Lent and Wygodzinsky 1979). Many species are competent vectors of Chagas disease transmitting *Trypanosoma cruzi* (Chagas, 1909) in their feces (Lent and Wygodzinsky 1979; Bern et al. 2011). Chagas disease is one of the ten most seriously neglected tropical diseases, which are currently estimated to affect nine million people, with more than 70 million people living under a serious risk of infection (Justi and Galvão 2017; WHO 2019).

The tribe Rhodniini currently contains two genera, *Rhodnius* Stål, 1859 (with 20 species) and *Psammolestes* Bergroth, 1911 (with three species) (Justi and Galvão et al. 2017; Rosa et al. 2017a; Nascimento et al. 2019). The main characters which distinguish *Rhodnius* and *Psammolestes* from the other genera of Triatominae are that their antenniferous tubercles do not close to eyes and the presence of callosities behind their eyes (Lent and Wygodzinsky 1979). *Rhodnius* is widely distributed in the Neotropical Region, and some species are the key vectors of Chagas disease in their respective ranges. *Rhodnius ecuadoriensis* Lent & León, 1958, for example, is one of the most important vector species of Chagas disease in Ecuador (Grijalva et al. 2015); *R. robustus* Larrousse, 1927 and *R. pictipes* Stål, 1872 are the vectors that cause public health problem in French Guiana (Barnabé et al. 2018). Most species of *Rhodnius* are arboreal, and their microhabitat preference patterns range from species that appear to inhabit a single species of palms (e.g., *R. brethesi* Matta, 1919 in *Leopoldinia piassaba*) to species that are found across several genera of palms (e.g., *R. pictipes* in *Attalea butyracea* and *Oenocarpus bataua*) (Lent and Wygodzinsky 1979; Barrett 1991; Carcavallo et al. 1998; Abad-Franch et al. 2005). *Rhodnius* is usually divided into three species groups, namely the *pictipes*, *prolixus*, and *pallescens* groups. *Pictipes* group includes six species, i.e., *R. amazonicus* Almeida, Santos & Sposina, 1973, *R. brethesi*, *R. paraensis* Sherlock, Guitton & Miles, 1977, *R. pictipes* Stål, 1872, *R. stali* Lent, Jurberg & Galvão,1993 and *R. zeledoni* Jurberg, Rocha & Galvão, 2009. *Prolixus* group includes eleven species, i.e., *R. barretti* Abad-Franch, Palomeque & Monteiro, 2013, *R. dalessandroi* Carcavallo & Barreto, 1976, *R. domesticus* Neiva & Pinto, 1923, *R. milesi* Carcavallo, Rocha, Galvão & Jurberg, 2001, *R. marabaensis*Souza et al., 2016, *R. montenegrensis*Rosa et al., 2012, *R. nasutus* Stål, 1859, *R. neglectus* Lent, 1954, *R. neivai* Lent, 1953, *R. prolixus* Stål, 1859, and *R. robustus*. *Pallescens* group includes three species, i.e., *R. colombiensis* Moreno Mejía, Galvão & Jurberg, 1999, *R. ecuadoriensis*, *R. pallescens* (Justi and Galvão 2017). These three species groups are currently recognized based on molecular data, distribution patterns, and morphometric analysis, and but not on qualitative morphological characters in the published literature (Dujardin et al. 1999; Lyman et al. 1999; Schofield and Dujardin 1999; Justi and Galvão 2017). The *pallescens* group is distributed to west of the Andes, whereas the *pictipes* and *prolixus* groups are mainly recorded to the east of the Andes (Abad-Franch and Monteiro 2007; Abad-Franch et al. 2009; Hernández et al. 2020).

The latest taxonomic revision of the entire genus was published approximately 40 years ago in the monograph on Triatominae by Lent and Wygodzinsky (1979) and contained descriptions of 11 of the 13 known species at that time. They regarded *R. amazonicus* as a synonym of *R. pictipes*, omitting *R. dalessandroi* because they were unable to examine specimens of this species. Bérenger and Pluot-Sigwalt (2002) and Rosa et al. (2017) made comparative studies between *R. pictipes* and *R. amazonicus* to prove the validity of *R. amazonicus*. The remaining seven species now included in *Rhodnius* were described after Lent and Wygodzinsky’s (1979) monograph (Lent et al. 1993a; Mejia et al. 1999; Valente et al. 2001; Jurberg et al. 2009; Rosa et al. 2012; Abad-Franch et al. 2013; Souza et al. 2016). *Rhodnius taquarussuensis*Rosa et al., 2017a was described as a new species but is now considered a phenotypic form of *R. neglectus* instead of a distinct species (Nascimento et al. 2019). Bérenger and Pluot-Sigwalt (2002) published a key for the *pictipes* group and Galvão (2014) released a key in Portuguese which included 12 *Rhodnius* species.

*Rhodnius* is relatively easy to distinguish from other Triatominae genera because of its long head and coloration pattern but shows low non-genitalic morphological variability between species in the genus, which may account for the difficulties in species identification. The female external genitalia was described for most species of the subfamily (Lent 1948; Abalos and Wygodzinsky 1951; Sherlock and Serafim 1967), but their diagnostic importance was dismissed in papers published by Lent and Jurberg (1968, 1969, 1975) which considered them uniform and, not useful for specific identification. The resurrection of female genitalia, as an important taxonomic tool, was attributed to Rosa et al. (2010) through a detailed study by scanning electron microscopy. Subsequently, several studies corroborate the diagnostic value of female genitalia (Rosa et al. 2012, 2014, 2017b; Rodrigues et al. 2018). The male external genitalia are usually used for generic and specific differentiation in assassin bugs. All published species except *R. barretti* had been documented with the external male genitalia. However, most of these descriptions were restricted to describing or comparing the shapes of the median process of pygophore (Lent and Wygodzinsky 1979; Harry 1993; Lent et al. 1993a; Mejia et al. 1999; Valente et al. 2001; Rosa et al. 2012, 2017a, b; Souza et al. 2016). Six species (*R. zeledoni*, *R. marabaensis*, *R. milesi*, *R. montenegrensis*, *R. stali*, and *R. colombiensis*) had only detailed illustrations of non-everted phalli, thus restricting the possibility of comparison various structures on the phallosoma and endosoma, which may be helpful in species-level identifications (Lent et al. 1993a; Mejia et al. 1999; Valente et al. 2001; Rosa et al. 2012, 2017b; Zhao et at. 2015; Souza et al. 2016). Drawings of endosomal structures that show the individual sclerites rather than the complete everted endosoma were published for only three species, *R. stali*, *R. pictipes*, and *R. milesi* (Lent et al. 1993a; Valente et al. 2001).

When examining the specimens of *Rhodnius*, two specimens from Bolivia were distinctly different from any other species found. In this study, they are named *Rhodnius micki* sp. nov. and described. Male genitalia are important in identifying assassin bugs, especially for *Rhodnius* which has low non-genitalic morphological variability between species. Therefore, special emphasis is put on their everted phallus, allowing for detailed photographic documentation of the phallus, particularly the sclerites of the endosoma. The diagnosis of the new species takes advantage of qualitative morphological features including genitalic features, and of geometric morphometric approaches to better characterize head and forewing shapes. Combining morphometric characters with distribution, we propose that this new species should be classified in the *pictipes* group. We also provide a synopsis of genitalic terminology applied to Triatominae and offer preferred terms to facilitate future investigations into the homology of these structures across Triatominae and even Heteroptera.

## Materials and methods

### Specimens

Type specimens and an additional male specimen of *R. robustus* Larrousse, 1927 are deposited in The Natural History Museum (**NHMUK**), London, United Kingdom.

Specimens of *R. stali*, *R. pictipes*, *R. pallescens*, and *R. ecuadoriensis* which were used for the geometric analysis came from colonies reared at Fundação Oswaldo Cruz (FIOCRUZ) in Brazil and were deposited at Fundação Oswaldo Cruz (**FIOCRUZ**).

### Dissections and measurements

**Figures 1–6.**
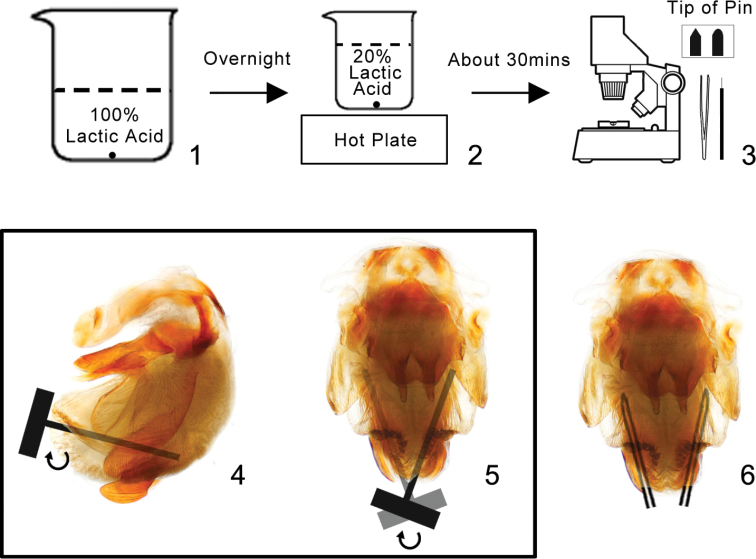
Process of dissection **1** soaking genitalia in 100% lactic acid overnight **2** boiling genitalia in 20% lactic acid solution for ~ 30 minutes **3–6** dissecting genitalia under microscope with forceps and blunted insect pin **4, 5** inserting the insect pin along the membrane of the endosoma and agitate the pin **6** using forceps to stretch the endosoma.

After softening the abdomens of dried specimens with wet tissue, the pygophores were removed and soaked in 100% lactic acid overnight (Fig. 1). They were then boiled in 20% lactic acid solution for ~ 30 minutes to remove muscles (Fig. 2). Dissections were carried out in the lactic acid under a Motic binocular dissection microscope. At this point, the endosoma was gently stretched with a pair of forceps (Ideal-Tek SS.SA) and insect pins (0#). The tip of the pins should be blunt (Fig. 3). At first, we inserted the insect pin along the membrane of the endosoma from the opening where the endosoma is everting out, and then gently agitated the pin along the membrane from one side to the other to make the phallosoma loose and make the endosoma move towards the tip of phallosoma, so that the opening is big enough and the forceps would enable to touch the sclerites of endosoma without breaking the membrane (Figs 4, 5). Forceps were used to grasp the sclerite and to stretch the endosoma (Fig. 6). After taking the photographs and other procedures, the dissected genitalia were preserved in glycerol in plastic tubes which were pinned under the corresponding specimens. Measurements were made using a calibrated micrometer and given in millimeters.

### Terminology

Because of the inconsistent use of terminology in Triatominae, after reviewing many taxonomic and comparative morphology papers of assassin bugs, the terminology adopted in this paper are listed in Table 1. It includes the preferred terms, definition of terms, previously used terms, and references.

### Images and image processing

Habitus images were obtained using a Canon EOS 7D and 60mm macro lens. Detail images of heads, pronota, and wings were obtained using a Microscope (Nikon SMZ18) with a Canon EOS 600D. Genital images were taken using an Olympus BX51 with a Canon EOS 450D. Images were stacked using the EOS Utility 2, and Helicon focus 5.3. Photographs were edited with Adobe Photoshop CS4, including adjustment of background color and cropping without modifying any characters. All the images were taken in the laboratory by the authors. The plate of male genitalia is that of the paratype.

### Morphometrics

In total, 42 specimens of five species, *R. ecuadoriensis* (ten specimens), *R. pallescens* (ten specimens), *R. pictipes* (ten specimens), *R. stali* (ten specimens), and *R. micki* sp. nov. (two specimens), were used in the analysis. and nine anatomical landmarks were extracted respectively on the heads and forewings. Thirteen landmarks of head (type II points, which combine geometric and biological or histological descriptions) (Gurgel-Gonçalves et al. 2008; Oliveira et al. 2017), and nine landmarks of wings (type I points, which homology comes from unique patterns in biological form) (Gurgel-Gonçalves et al. 2008; Feliciangeli et al. 2007; Costa et al. 2009; Oliveira et al. 2017) were extracted based on the landmarks used in previous works. These landmarks were digitized with tpsUtil 1.46 (Rohlf 2010) and tpsdig2 v.2.16 (Rohlf 2008). To quantify the shape variation related with the shape dimensions, the digitized data were analyzed using morphoJ 1.06d (Klingenberg 2011). Variability in the shape space was assessed using a Principal Component Analysis (PCA). To better visualize the shape variation, thin plate spline visualization was used to get the average shapes of these characters.

## Taxonomy

### Reduviidae Latreille, 1807

#### Triatominae Jeannel, 1919

##### Rhodnius

Taxon classificationAnimaliaHemipteraReduviidae

Stål, 1859

2990C28C-AB54-56CE-923A-26B6E2B5D928

###### Type of genus.

*Rhodnius prolixus* Stål, 1859.

##### Rhodnius micki sp. nov.

Taxon classificationAnimaliaHemipteraReduviidae

75283164-70B8-5A2F-AAC2-B8778B463A42

http://zoobank.org/226A56E5-FDF8-4850-9426-80B3C4D79FC5

###### Type materials.

Bolivia: Santa Cruz, Saavedra, C.J. Pruett [leg.], 1 male holotype, 10.v.1989, 1 male paratype, 1.iii.1989 (NMHUK).

###### Diagnosis.

General coloration dark brown. Head relatively short, only slightly longer than the pronotum. Eyes small, width of the eye shorter than the synthlipsis. Central area of the anterior lobe of the pronotum conspicuously dark and its humeral angle of the posterior lobe relatively sharply curved. Legs brown. The median process of the pygophore long and bifid on the tip. The medial basal sclerite of the phallosoma with two straight and flat projections. One distal dorsal sclerite of the endosoma bifurcated, and its tip rounded and curved slightly inward.

###### Description.

**Figures 7–11.**
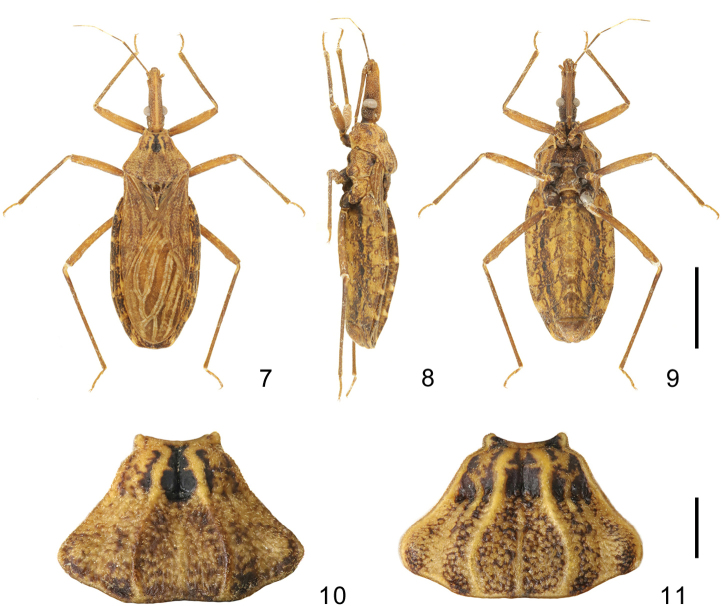
**7–9** holotype of *Rhodnius micki* sp. nov. **11***Rhodnius stali* Lent, Jurberg & Galvão, 1993 **7** dorsal side **8** lateral side **9** ventral side **10, 11** pronotum. Scale bars: 5.00 mm (**7–9**); 1.00 mm (**10, 11**)

***Coloration*.** Body generally dark brown. Head with light median longitudinal stripe extending from the apex of clypeus to the posterior portion of ocelli; eyes blackish; middle of third segment and posterior half of forth segment yellow; a pair of black stripes on the dorsal surface of neck, half of lateral side and ventral side dark. Pronotum with a pair of submedian carinae and lateral margin yellow; concave areas on anterior lobe, especially the central area darkened; posterior lobe dark with scattered irregular small yellow spots. Scutellum dark with a yellow “Y”-shaped ridge; the tip of scutellar process white. Hemelytra generally brown and mottled; corium with small lightly colored spots; membrane with narrowly rimmed pale-yellow veins, area between veins with scattered light color spots. Legs mottled with yellow spots; tarsi yellowish (Fig. 7). Connexivum dark and mottled with yellow spots, posterior one fourth of every segment almost yellow; ventral surface of the abdomen yellowish with scattered irregular dark brown spot; sternites light brown to black, with irregular dark brown spots, center of sternite II and a pair of sublateral elliptical spots of each segment dark (Fig. 9); spiracles with a brown narrowly margin (Fig. 8).

###### Structure.

**Figures 12–16.**
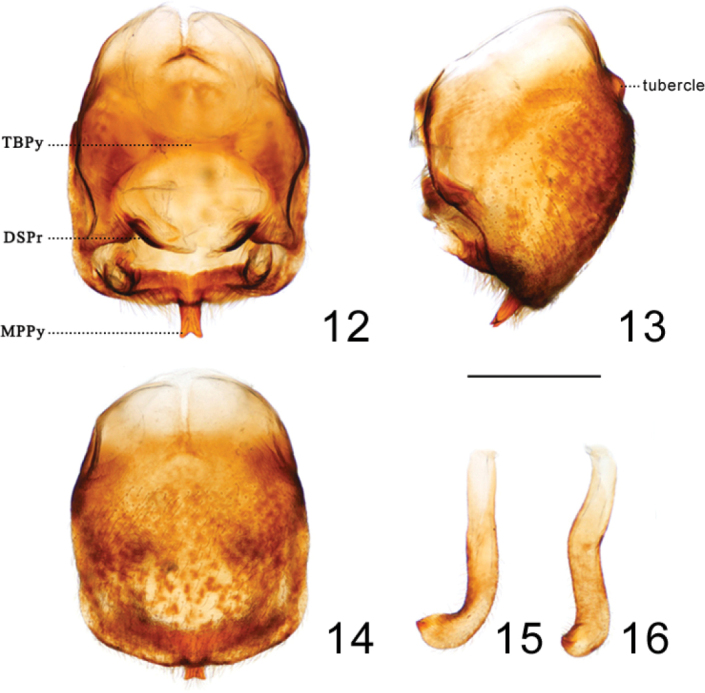
Pygophore and paramere of paratype of *Rhodnius micki* sp. nov. **12–14** pygophore **12** dorsal view **13** lateral view **14** ventral view **15, 16** paramere: **15** dorsal view **16** lateral view. Scale bars: 1.00 mm. Abbreviations: **DSPr** dorsal sclerites of pygophore **MPPy** Median process of pygophore **TBPy** Transverse bridge of pygophore.

**Figures 17–26.**
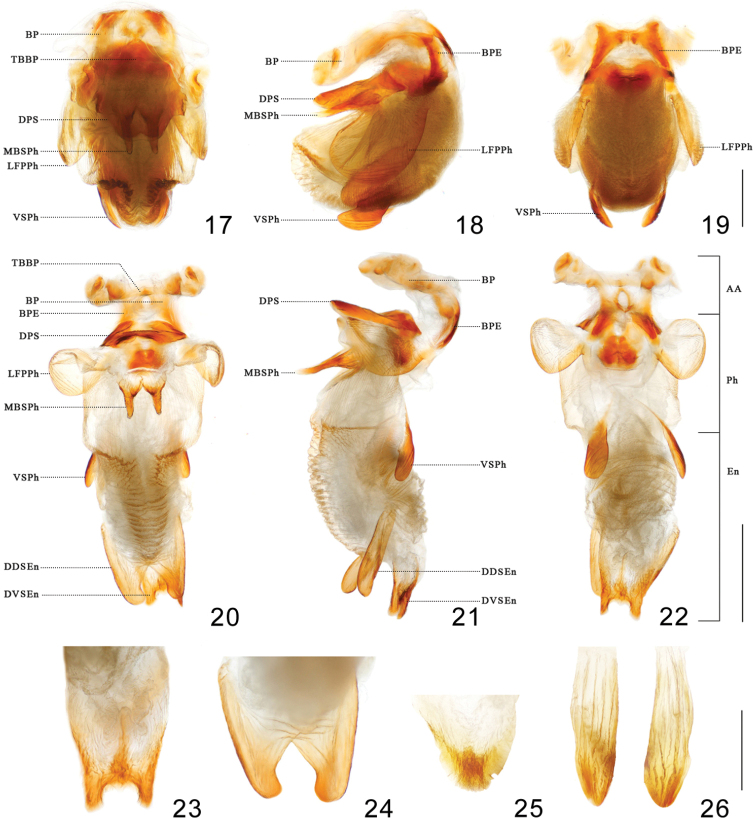
Pallus **17–24***Rhodnius micki* sp. nov. **25, 26***Rhodnius robustus***17–19** non-everted phallus **20–26** everted phallus **17, 20** dorsal side **18, 21** lateral side **19, 22** ventral side **23, 25** distal ventral sclerite of endosoma **24, 26** distal dorsal sclerite of endosoma. Scale bars: 1.00 mm (**11–16**); 0.50 mm (**17–20**). Abbreviations: **BP** basal plate **TBBP** transverse bridge of basal plate **DPS** dorsal phallothecal sclerite **MBSPh** medial basal sclerite of phallosoma **LFPPh** lateral flat-like prolongation of phallosoma **VSPh** ventral sclerite of phallosoma **BPE** basal plate extension **DDSEn** distal dorsal sclerite of endosoma **DVSEn** distal ventral sclerite of the endosoma **AA** articulatory apparatus **Ph** phallosoma **En** endosoma.

***Head*.** Elongated and granulose, almost 2.5 × as long as width across eyes (1:2.6–2.59), slightly longer than length of pronotum (1:1.17–1.21); apex of maxillary plate surpassing clypeus; anteocular region ~ 3 × as long as postocular region in length (1: 2.84–3.15); eyes small, width of eye in dorsal view shorter than synthlipsis (1:0.60); in lateral view, eyes far away from upper surface of head and approaching to lower surface; ratio of antennal segments 1:5.11–6.29:4.66–5.14:3.55–4.43; first labial segment proceeding toward antenniferous tubercle and second labial segment approaching to posterior margin of head. Ratio of labial segments 1:2.78–3.13:0.61–0.83. ***Thorax*.** Anterolateral angles triangle-like. Surface of pronotum granulose, length of posterior pronotal lobe ~ 2 × as that of anterior lobe (1:1.89–1.93); posterior pronotal lobe ~ 1.5 ×as wide as anterior lobe (1:1.52–1.74); median longitudinal furrow of anterior lobe deep on the median transverse furrow; humeral angles sharply curved relatively to other species of ***Rhodnius*** (Fig. 10). Scutellum triangular with a yellow Y-shaped ridge; subapical portion with a cone-shaped process. Pleura of meso- and metathoraxes winkled. Legs long and slender. Hemelytra approaching tip of abdomen. ***Male genitalia*** (Figs 12–26). Pygophore (Figs 12–14) globular with a tubercle on the bottom of the ventral surface (Fig. 13); transverse bridge of pygophore (TBPy) strongly sclerotized and narrow; a pair of dorsal sclerites of genital opening (DSPr) large; median process of pygophore (MPPy) long, bifid at apical portion and tilting 45 degrees to the dorsal side in lateral view. Parameres (Figs 15, 16) strongly curved at apex and with a denticle. Basal plate (BP) hexagonal in dorsal view, diameter of the arms similar to that of the transverse bridge of basal plate (TBBP) (Fig. 17); basal plate extension (BPE) short and approximately half shorter to arms of basal plate in length (Figs 18, 21); dorsal phallothecal sclerite (DPS) flat, as a subrectangular with round angles; medial basal sclerite of phallosoma (MBSPh) bifid with two straight and flat projections (Figs 17, 18, 20, 21), and both of them slightly swelled at base; lateral flap-like prolongation of phallosoma (LFPPh) large (Figs 17–22); two ventral sclerites of phallosoma (VSPh) elongated ovoid (Figs 17–22); the tip of non-everted phallus slightly sclerotized on the dorsal and lateral surface, and the surface of the phallosoma with indistinct stripes (Figs 17, 18); distal dorsal sclerite of endosoma (DDSEn) bifurcated, tips rounded, and curved inward lightly (Figs 20, 21, 24); distal ventral sclerite of the endosoma (DVSEn) smaller than the dorsal sclerite and bifurcated with two projections set far apart (Figs 21–23). The membrane of endosoma on the dorsal surface wrinkled and a bit thicker than other part of membrane.

###### Etymology.

The species epithet is named in honor of Mr. Mick Webb (NHMUK), who had helped us in many ways in the study of Hemiptera.

###### Measurements.

[in mm, ♂ (n = 2)] Total length to tip of abdomen 17.20–17.33. Length of head (exclude neck) 3.21–3.55; width of head 1.40–1.43; length of anteocular 2.27–2.30; length of postocular 0.73–0.80; width of eye 0.40–0.44; length of synthlipsis 0.67–0.73. Length of antennal segments I–IV=0.35–0.45/2.20–2.30/1.80–2.10/1.55–1.60; length of visible labial segments I–III=0.80–0.90/2.50/0.60. Length of anterior lobe of pronotum 0.90–0.93; length of posterior pronotal lobe 1.70–1.93; width of anterior pronotal lobe 2.30–2.33; width of posterior pronotal lobe 4.00–4.15. Length of scutellum 1.70–1.75; width of scutellum 1.80–1.90; length of hemelytron 10.40–10.50. Width of abdomen 5.35–5.40. (all the former numbers are for holotype, except length of total, anteocular, second and fourth segment, and width of abdomen).

###### Additional material.

*Rhodnius robustus* Larrousse, 1927 (1♂, Brazil: Belém, Instituto Evandro Chagas, reared in lab, 20.II.1992) (NHMUK).

###### Geometric morphometrics

**Figures 27, 28.**
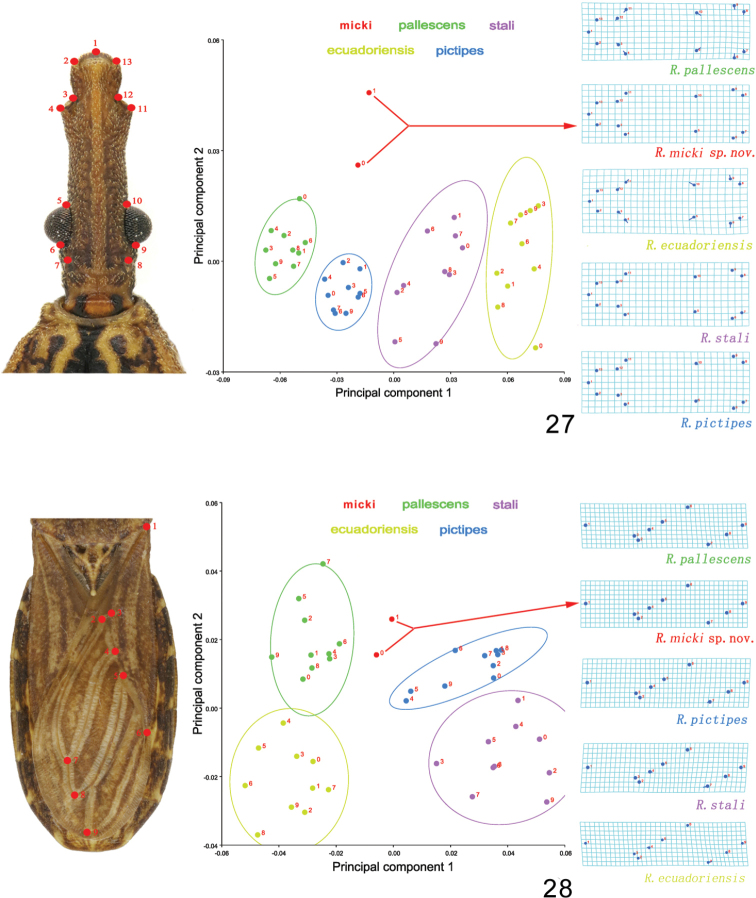
Morphological variations of five *Rhodnius* species based on Principal Component Analysis. The 90% equal frequency ellipses containing approximately 90% of the data points are shown. The thin-plate splines show the average shape for each species, corresponding to the deformation of the landmarks compared with the origin (the average shape of all species) **27** head **28** fore wing.

(Figs 27, 28) On the one hand, *R. pictipes* and *R. stali* appear to be the most morphologically similar species to *R. micki* sp. nov. having relatively short head, only slightly longer than the pronotum, and a defined transverse sulcus on their pronotum. On the other hand, *Rhodnius ecuadoriensis*, *R. pallescens*, *and R. micki* sp. nov. do not have dark rings on the tibiae which is a significant diagnostic character of *Rhodnius*. Based on the morphometrics of the head and the particular coloration of the legs, we compared before mentioned four species to *R. micki* sp. nov. For head shape analysis (Fig. 27), the contribution of the first principal (PC1) component accounted for 81.79% of the total variation, whereas the second principal component (PC2) accounted for 6.26%. In the factorial map, five species were separated. The type specimens of *R. micki* sp. nov. were far away from the others. The thin plate spline visualization showed that the fifth and tenth landmarks located on the anterior margin of eye contributed most to the shape difference among these species. The size of the eye and the length of the anteocular and postocular regions might be the most significant differences among them. For wing vein analysis (Fig. 28), the contribution of the first principal (PC1) component accounted for 58.46% of the total variation and the second principal component (PC2) accounted for 22.21%. The points of *R. micki* sp. nov. were also distinct from those of the other four species, and these four species were separated from each other too. The thin plate spline visualization showed that the seventh landmark contributed most to the shape difference among these species. It implied that the position of the intersection of the Cu and An1 veins may be the most variable among them.

## Discussion

### Comparison with other species

It is relatively easy to distinguish this species from other *Rhodnius* species because of its relatively sharply curved humeral angles and unique color pattern. *Rhodnius stali* and *R. pictipes* are similar to *R. micki* sp. nov. because their heads are all relatively short, only slightly longer than their pronota, and their pronota have a defined transverse sulcus. However, the tibiae of *R. micki* sp. nov. are uniformly dark brown, the humeral angle is sharply curved (Fig. 10), and the third antennal segment is black, whereas the other two both have a distinct dark ring on each tibia, only the anterior half of the third antennal segment is black, and the humeral angle is broadly rounded (Fig. 11). *Rhodnius ecuadoriensis* and *R. pallescens* do not have any tibial rings. *Rhodnius ecuadoriensis* is smaller than *R. micki* sp. nov., and the head of *R. pallescens* is obviously longer than the pronotum. *Rhodnius micki* sp. nov. is darker and its submedian carinae on the posterior lobe are not obvious; the posterior quarter of every connexival segment on the dorsal side is yellow. Differences between *R. micki* sp. nov. and the other species in the male genitalia are significant. The median processes of the pygophore of *R. micki* sp. nov., *R. stali*, and *R. pictipes* are bifid, but the former one is bifid at its tip, with small projections, whereas the median processes of the pygophores of *R. stali* and *R. pictipes* are bifid (Lent et al. 1993a) at the base with long projections, and those of *R. ecuadoriensis* and *R. pallescens* are not bifid (Lent and Wygodzinsky 1979; Mejia et al. 1999). The parameres of *R. micki* sp. nov. are narrower than those of *R. stali* and *R. pictipes* (Lent et al. 1993). The medial basal sclerite of its phallosoma (MBSPh) is bifid with two flat and straight arms; other *Rhodnius* species do not have a medial basal sclerite or it is not bifid (Y. Zhao unpublished data). The distal ventral sclerite of the endosoma (DVSEn) of *R. micki* sp. nov. is smaller and less sclerotized than those in *R. stali* and *R. pictipes* (Lent et al. 1993a), and the distal dorsal sclerite (DDSEn) is bifurcated deeply, curved inward, and more heavily sclerotized than *R. ecuadoriensis* and *R. pallescens* (our unpublished data). Therefore, Genitalic structures, especially distal ventral sclerite of the endosoma (DVSEn) and distal dorsal sclerite of the endosoma (DDSEn), can provide more information to fully compare the species of *Rhodnius*. According to geometric morphological analysis, *R. micki* sp. nov. is relatively isolated on the factorial map, which suggests that the *R. micki* sp. nov. is also distinguished from those species relatively easily based on the shapes of the head and wing.

### Species group assignment

*Rhodnius micki* sp nov. is known from Santa Cruz, Bolivia, where some species of *pictipes* group and *prolixus* group, i.e., *R. stali*, *R. pictipes*, and *R. robustus* are distributed (Chávez 2006; Schofield and Galvão 2009; Justi et al. 2010; Soto-Vivas et al. 2018). *Rhodnius stali* and *R. pictipes*, which are the most similar species to *R. micki* sp. nov. based on the non-genitalic characters mentioned above, both belong to the *pictipes* group. With respect to genitalic characters, they are also similar because they all have a single distal dorsal sclerite on the endosoma (Lent et al. 1993a). Based on our observations (unpublished), species in the *prolixus* group, such as *R. robustus*, have two symmetrical sclerites located in the same position, and the shape of the ventral sclerite of endosoma is triangle (Figs 25, 26). Therefore, we infer that *R. micki* sp. nov. should be included in the *pictipes* group based on distribution and genitalic characters.

### Terminology of morphological characters

Historically, the terminology of Triatominae, especially male genitalic terms, has developed at least partially in isolation from that of Reduviidae. A plethora of terms have been used for homologous genitalic structures, and in some cases different structures have used the same name. This inconsistency results in incompatible and sometimes misleading terminology for taxonomic descriptions and diagnoses. For example, some researchers have variously used the terms aedeagus, phallus, phallosoma, conjunctiva and phallothecal plate when describing the apical apart of the intromittent organ, and the sclerotized plate beneath the basal plate (Lent and Wygodzinsky 1979; Mejia et al. 1999; Valente et al. 2001; Jurberg et al. 2009; Rosa et al. 2012; Gil-Santana and Galvão 2013; Souza et al. 2016; Oliveira et al. 2018). To avoid ambiguity and achieve consistency with the description of other assassin bugs, we adopt the following terms in this study. Male genitalia consist of pygophore, parameres, and phallus. The articulatory apparatus is composed of basal plate, basal plate bridge, and basal plate extension. The dorsal phallothecal sclerite (DPS) is regarded as the dorsal part of phallosoma. To clarify each sclerite’s position, we rename these sclerites with adjectives describing their position, while being as consistent as possible with previous terms. We adopt medial basal sclerite of phallosoma to denote the sclerite on the dorsal side of phallosoma. Two pairs of sclerites on the lateral and ventral sides of phallosoma are called lateral flat-like prolongation of phallosoma (LFPPh) and ventral sclerite of phallosoma (VSPh) respectively. Sclerites at the tip of the endosoma are renamed distal dorsal sclerite of endosoma (DDSEn) and distal ventral sclerite of endosoma (DVSEn). All the preferred terms and synonyms are shown in Table 1.

**Table 1.** Terminology used in this study with synonyms from the literature.

Preferred term (abbreviation)	Definition	Previously used terms	References
Articulatory apparatus (AA)	System of plates and apodemes for suspension of phallus and attachment of its motor muscles	Articulatory apparatus (Apb) (apt)	Lent and Wygodzinsky 1979; Lent and Jurberg 1984; Lent and Jurberg 1987; Lent et al. 1993b; Mejia et al. 1999; Carcavallo et al. 2001; Valente et al. 2001; Sandoval et al. 2007; Jurberg et al. 2009; Forero et al. 2010; Berniker et al. 2011; Forero and Weirauch 2012; Gil-Santana and Galvão 2013; Jurberg et al. 2013; Castro-Huertas and Forero 2014; Gil-Santana 2017; Chłond et al. 2018
		Phallobase	Zhao et al. 2015
Basal plate (BP)	Paired major plates of articulatory apparatus	Basal plate (Plb)	Lent and Wygodzinsky 1979; Lent and Jurberg 1984; Lent and Jurberg 1987; Lent et al. 1993a; Mejia et al. 1999; Carcavallo et al. 2001; Valente et al. 2001; Cai and Tomokuni 2003; Weirauch 2008; Sandoval et al. 2007; Jurberg et al. 2009; Frías-Lasserre 2010; Forero et al. 2010; Berniker et al. 2011; Forero and Weirauch 2012; Rosa et al. 2012; Gonçalves et al. 2013; Jurberg et al. 2013; Zhao et al. 2015; Ishikawa and Naka 2016; Souza et al. 2016; Rosa et al. 2017a; Chłond et al. 2018; Oliveira et al. 2018
		Basal plate arm (bpa)	Gil-Santana 2017
		Basal arm	Gil-Santana and Galvão 2013
Basal plate extension (BPE)	Ventral sclerite arising from the basal plate	Basal plate extension (bpe)	Weirauch 2008; Berniker et al. 2011; Forero and Weirauch 2012; Oliveira et al. 2018
		Pedicel (ped) (pd)	Cai and Tomokuni 2003; Gil-Santana and Galvão 2013; Castro-Huertas and Forero 2014; Zhao et al. 2015; Gil-Santana 2017
		Median (Medium) extension of the basal plate (EPlb) (MeBp)	Lent and Jurberg 1984; Lent and Jurberg 1987; Lent et al. 1993a, b; Mejia et al. 1999; Carcavallo et al. 2001; Valente et al. 2001; Sandoval et al. 2007; Jurberg et al. 2009; Frías-Lasserre 2010; Rosa et al. 2012; Souza et al. 2016; Rosa et al. 2017a
		Median basal plate	Gonçalves et al. 2013
		Plate extension (pext)	Forero et al. 2010
Distal dorsal sclerite of endosoma (DDSEn)	Paired or single sclerite on the tip of endosoma which is on the dorsal side of the distal ventral sclerite	Process of endosoma	Jurberg et al. 2009
		Processes of endosoma 1 (PrEn 1)	Valente et al. 2001
		Processes of endosoma 2 (PrEn 2)	Lent et al. 1993a; Mejia et al. 1999
Distal ventral sclerite of endosoma (DVSEn)	A single sclerite on the tip of endosoma which is on the ventral side of the distal ventral sclerite	Processes of endosoma	Jurberg et al. 2009
		Processes of endosoma 1 (PrEn 1)	Lent et al. 1993a; Mejia et al. 1999
		Processes of endosoma 2 (PrEn 2)	Valente et al. 2001
Dorsal phallothecal sclerite (DPS)	Sclerotized proximal part of phallosoma	Dorsal phallothecal sclerite (dps)	Cai and Tomokuni 2003; Weirauch 2008; Forero et al. 2010; Berniker et al. 2011; Forero and Weirauch 2012; Castro-Huertas and Forero 2014; Zhao et al. 2015; Ishikawa and Naka 2016; Gil-Santana 2017; Chłond et al. 2018; Lapischies et al. 2019
		Phallosoma (Ph)	Lent and Jurberg 1984; Lent and Jurberg 1987; Lent et al. 1993a, b; Mejia et al. 1999; Carcavallo et al. 2001; Valente et al. 2001; Sandoval et al. 2007; Jurberg et al. 2009; Rosa et al. 2012; Gonçalves et al. 2013; Jurberg et al. 2013; Souza et al. 2016; Rosa et al. 2017a, b; Oliveira et al. 2018
		Dorsal phallotheca plate	Lent and Wygodzinsky 1979; Gil-Santana and Galvão 2013
		Phallotheca plate	Frías-Lasserre 2010
Dorsal sclerites of pygophore (DSPr)	Posterior dorsal sclerotization of pygophore	Dorsal sclerotization of genital opening, tergite 9 (t9)	Forero and Weirauch 2012
Endosoma (En)	Distal portion of phallus which can be reverted	Endosoma	Lent and Wygodzinsky 1979; Lent and Jurberg 1984; Lent and Jurberg 1987; Lent et al. 1993a, b; Mejia et al. 1999; Carcavallo et al. 2001; Valente et al. 2001; Cai and Tomokuni 2003; Jurberg et al. 2009; Frías-Lasserre 2010; Forero and Weirauch 2012; Jurberg et al. 2013; Castro-Huertas and Forero 2014; Zhao et al. 2015; Ishikawa and Naka 2016; Souza et al. 2016; Rosa et al. 2017a; Oliveira et al. 2018; Lapischies et al. 2019
Lateral flap-like prolongation of phallosoma (LFPPh)	Paired of sclerite on the lateral side of phallosoma	Lateral flat-like prolongation of the phallosoma	Forero and Weirauch 2012
		Processes of the conjunctiva 1 (PrCj 1)	Lent et al. 1993a; Mejia et al. 1999; Valente et al. 2001
		Processes of the conjunctiva	Jurberg et al. 2009
Mandibular plate	Laterad of clypeus and dorsad of maxillary plate	Mandibular plate	Weirauch 2008; Berniker et al. 2011; Ishikawa and Naka 2016
		Jugum	Lent and Wygodzinsky 1979; Lent et al. 1993a; Carcavallo et al. 2001; Gonçalves et al. 2013; Souza et al. 2016
Maxillary pate	Ventral to mandibular plate	Maxillary plate	Weirauch 2008; Berniker et al. 2011; Castro-Huertas and Forero 2014; Ishikawa and Naka 2016
		Gena (ge)	Lent and Wygodzinsky 1979; Lent et al. 1993a; Carcavallo et al. 2001; Sandoval et al. 2007; Jurberg et al. 2009; Gonçalves et al. 2013; Souza et al. 2016; Rosa et al. 2017a; Chłond et al. 2018; Oliveira et al. 2018
Medial basal sclerite of phallosoma (MBSPh)	Basal part of a phallosoma, often sclerotized	Vesica (V)	Lent and Wygodzinsky 1979; Lent and Jurberg 1987; Carcavallo et al. 2001; Cai and Tomokuni 2003; Sandoval et al. 2007; Gonçalves et al. 2013; Jurberg et al. 2013
		Median distal process	Gil-Santana and Galvão 2013
		Median process of endosoma	Gil-santana 2014
		Central sclerite of endosoma (cs)	Lapischies et al. 2019
		Median basal sclerotization (mbs)	Forero et al. 2010; Berniker et al. 2011
		Processes of conjunctiva 2	Lent et al. 1993a; Mejia et al. 1999
		Dorsobasal large sclerite	Ishikawa et al. 2007
Median process of pygophore (MPPy)		Median process of (the) pygophore (PrP)	Lent and Wygodzinsky 1979; Lent and Jurberg 1984; Lent et al. 1993a, b; Mejia et al. 1999; Carcavallo et al. 2001; Valente et al. 2001; Sandoval et al. 2007; Jurberg et al. 2009; Forero et al. 2010; Rosa et al. 2012; Gil-Santana and Galvão 2013; Castro-Huertas and Forero 2014; Souza et al. 2016; Rosa et al. 2017a; Oliveira et al. 2018
		Median pygophore process	Cai and Tomokuni 2003; Zhao et al. 2015
Phallosoma (Ph)	Proximal portion of phallus, between basal plate and endosoma.	Phallosoma	Lent and Wygodzinsky 1979; Forero and Weirauch 2012; Castro-Huertas and Forero 2014; Zhao et al. 2015
		Conjunctive	Lent and Jurberg 1984; Lent and Jurberg 1987; Lent et al. 1993a, b; Mejia et al. 1999; Carcavallo et al. 2001; Valente et al. 2001; Sandoval et al. 2007; Jurberg et al. 2009; Rosa et al. 2012; Gonçalves et al. 2013; Souza et al. 2016; Rosa et al. 2017a
Phallus (P)	Intromittent organ inside the pygophore	Phallus (Ph) (P)	Lent and Jurberg 1984; Lent and Jurberg 1987; Lent et al. 1993a, b; Mejia et al. 1999; Carcavallo et al. 2001; Valente et al. 2001; Cai and Tomokuni 2003; Ishikawa et al. 2007; Sandoval et al. 2007; Weirauch 2008; Jurberg et al. 2009; Frías-Lasserre 2010; Forero et al. 2010; Forero and Weirauch 2012; Rosa et al. 2012; Gonçalves et al. 2013; Gil-Santana and Galvão 2013; Jurberg et al. 2013; Castro-Huertas and Forero 2014; Zhao et al. 2015; Ishikawa and Naka 2016; Souza et al. 2016; Rosa et al. 2017a; Gil-Santana 2017; Oliveira et al. 2018; Lapischies et al. 2019
		Aedeagus	Lent and Wygodzinsky 1979
Struts	Paired sclerites on the ventral side of dorsal phallothecal sclerite	Struts (str)	Lent and Wygodzinsky 1979; Cai and Tomokuni 2003; Forero et al. 2010; Gonçalves et al. 2013; Gil-Santana and Galvão 2013; Zhao et al. 2015
		Phallosoma support (Sph)	Lent and Jurberg 1984; Lent and Jurberg 1987; Lent et al. 1993b; Carcavallo et al. 2001; Sandoval et al. 2007; Jurberg et al. 2013; Oliveira et al. 2018
		Struts of phallus	Ishikawa and Naka 2016
Synthlipsis	Minimum interocular distance	Synthlipsis	Lent and Wygodzinsky 1979; Valente et al. 2001; Sandoval et al. 2007; Jurberg et al. 2013; Zhao et al. 2015
		Interocular space	Ishikawa and Naka 2016; Gil-Santana 2017
		Interocular region	Valente et al. 2001; Jurberg et al. 2013
Transverse bridge of basal plate (TBBP)	Connection between two basal plate	Basal plate; Bridge (bpb)	Lent and Wygodzinsky 1979; Cai and Tomokuni 2003; Berniker et al. 2011; Castro-Huertas and Forero 2014; Ishikawa and Naka 2016; Gil-Santana 2017
		Basal bridge (PB)	Lent and Jurberg 1984; Lent and Jurberg 1987; Lent et al. 1993; Mejia et al. 1999; Carcavallo et al. 2001; Valente et al. 2001; Sandoval et al. 2007; Gonçalves et al. 2013; Gil-Santana and Galvão 2013; Jurberg et al. 2013
Transverse bridge of pygophore (TBPy)	Anterior dorsal sclerotization of pygophore	Transverse bridge of the pygophore (br)	Forero and Weirauch 2012; Castro-Huertas and Forero 2014
Ventral sclerite of phallosoma (VSPh)	Paired of sclerites on the ventral side of phallosoma	Processes of the conjunctiva 2 (PrCj 2)	Valente et al. 2001
		Processes of the conjunctiva (PrCj)	Lent et al. 1993b
		Processes of the conjunctiva 3 (PrCj 3)	Mejia et al. 1999

## Supplementary Material

XML Treatment for Rhodnius

XML Treatment for Rhodnius micki

## Supplementary materials

Supplementary material 1 TPS file of landmarks of *R. ecuadoriensis* Data type measurement Explanation note This TPS file contains the landmark cordinates of *R. ecuadoriensis*. https://binary.pensoft.net/file/501473This dataset is made available under the Open Database License (http://opendatacommons.org/licenses/odbl/1.0/). The Open Database License (ODbL) is a license agreement intended to allow users to freely share, modify, and use this Dataset while maintaining this same freedom for others, provided that the original source and author(s) are credited.Yisheng Zhao, Cleber Galvão, Wanzhi Cai

Supplementary material 2 TPS file of landmarks of *R. micki* Data type measurement Explanation note This TPS file contains the landmark cordinates of *R. micki*. https://binary.pensoft.net/file/501474This dataset is made available under the Open Database License (http://opendatacommons.org/licenses/odbl/1.0/). The Open Database License (ODbL) is a license agreement intended to allow users to freely share, modify, and use this Dataset while maintaining this same freedom for others, provided that the original source and author(s) are credited.Yisheng Zhao, Cleber Galvão, Wanzhi Cai

Supplementary material 3 TPS file of landmarks of *R. pallescens* Data type measurement Explanation note This TPS file contains the landmark cordinates of *R. pallescens*. https://binary.pensoft.net/file/501475This dataset is made available under the Open Database License (http://opendatacommons.org/licenses/odbl/1.0/). The Open Database License (ODbL) is a license agreement intended to allow users to freely share, modify, and use this Dataset while maintaining this same freedom for others, provided that the original source and author(s) are credited.Yisheng Zhao, Cleber Galvão, Wanzhi Cai

Supplementary material 4 TPS file of landmarks of *R. pictipes* Data type measurement Explanation note This TPS file contains the landmark cordinates of *R. pictipes*. https://binary.pensoft.net/file/501476This dataset is made available under the Open Database License (http://opendatacommons.org/licenses/odbl/1.0/). The Open Database License (ODbL) is a license agreement intended to allow users to freely share, modify, and use this Dataset while maintaining this same freedom for others, provided that the original source and author(s) are credited.Yisheng Zhao, Cleber Galvão, Wanzhi Cai

Supplementary material 5 TPS file of landmarks of *R. stali* Data type measurement Explanation note This TPS file contains the landmark cordinates of *R. stali*. https://binary.pensoft.net/file/501477This dataset is made available under the Open Database License (http://opendatacommons.org/licenses/odbl/1.0/). The Open Database License (ODbL) is a license agreement intended to allow users to freely share, modify, and use this Dataset while maintaining this same freedom for others, provided that the original source and author(s) are credited.Yisheng Zhao, Cleber Galvão, Wanzhi Cai
